# Syphilis of the Nervous System

**Published:** 1871-01

**Authors:** 


### Selections.

### SYPHILIS OF THE NERVOUS SYSTEM.

Dr. E. L. Keys read, at a recent meeting of the N. Y. Med. Jour. Association, portions of an extended and important paper upon this subject, based upon the clinical observations of thirty-four cases. It appears in full in the current number of the *New York Medical Journal.* We have space only for the summary of its conclusion:

1st. That nervous symptoms depending upon syphilis may arise within the first few weeks after contraction of an infecting chancre, or at any period later during the life of the individual.

2d. That it is presumable, from the study of published autopsies, that the earlier a nervous symptom (paralytic or otherwise) occurs, the less likely is there to be any material lesion which an autopsy can reveal; and that in a given case there exists no constancy of relation between the nature, the situation, and the severity of the lesion, and the nature, situation, and severity of the nervous symptom to which that lesion may give rise.

3d. That cerebral congestion is probably the pathology of many of the earlier nervous syphilitic symptoms.

4th. That syphilitic hemiplegia occurs, as a rule, without loss of consciousness even when the attack is sudden, but that the paralysis usually comes on gradually, the patient being under forty years of age, and having had fixed, constant headache for some time before the attack.

5th. That mydriasis existing alone or with other nervous symptoms, without positive disease of the eye, is presumptive evidence of syphilis.

6th. That paralysis of single muscles or sets of muscles are frequently syphilitic.

7th. That syphilitic paraplegia generally comes on gradually, often without any local symptom to call the patient's attention to the injured portion of the cord, and that is rarely complete. That the bladder always suffers more or less, and calls for special local treatment. That paraplegia may be developed as a symptom of inherited syphilis.

8th. That syphilitic epilepsy usually occurs after thirty in patients who have not had epilepsy in early life. That headache is liable to precede the attacks. That the convulsions occur often, many in quick succession, the intermission between the series of attacks being comparatively long; but that, during this period, headache or other nervous symptoms exist and become aggravated, contrary to what obtains in idiopathic epilepsy. That syphilitic epilepsy is liable to be associated with or followed by some form of paralysis.

9th. That aphasia is often associated with the intellectul disturbances caused by syphilis.

10th. That loss of memory is a common nervous symptom of syphilis, as are also all forms of mental disturbance, from mild hallucinations and illusions up to actual insanity, and all these without any necessary accompanying paralysis.

11th. That inordinate emotional expressions are often associated with the mental weakness caused by syphilis.

12th. That care must be taken to distinguish certain symptoms caused by gout from the same symptoms owing their origin to syphilis.

13th. That the prognosis is better, as a rule, for nervous symptoms caused by syphilis than for the same symptoms depending on a lesion equal in extent caused by another malady of the nervous centres; but that, after the arrest of the disease, an indellible impression is often left upon the nerve tissue, which manifests itself by impaired function, and which treatment cannot overcome.

14th. That the iodide of potassium pushed rapidly to toleration, unless the symptoms subside before that point is reached, is the main outline of treatment. That mercury used at the same time or alternated with the iodide of potassium is often of great value in protracted or inveterate cases, and that tonics, change of air and surroundings, frequently influence the effect of treatment in a marked degree, and may become essentials to success.—*Medical Record.*

